# Supplementary material for: Association of CYP26C1 Promoter Hypomethylation with Small Vessel Occlusion in Korean Subjects
Source: Genes (Basel). 2021 Oct 14;12(10):1622. doi: 10.3390/genes12101622 (PMC8535232; doi:10.3390/genes12101622)
Supplement: Supplementary file 1 [file genes-12-01622-s001.zip › genes-1403400-supplementary.pdf]

Supplementary Tables

Table S1. Different DNA methylation levels of each CpG site in the CYP26C1 promoter region between normal subjects and patients with SVO.

ID	Loci	Transcriptional start site	Normal	SVO	*p*-Value
CpG_2	94,820,183	-372	0.362 ±0.069	0.334 ±0.071[a]	0.0335[b]
CpG_3	94,820,189	-366	0.184 ±0.048	0.151 ±0.051	0.0001
CpG_4	94,820,205	-350	0.270 ±0.052	0.235 ±0.064	0.0004
CpG_5	94,820,209	-346	0.183 ±0.048	0.155 ±0.048	0.0005
CpG_6	94,820,231	-324	0.135 ±0.049	0.116 ±0.041	0.0136
CpG_7	94,820,236	-319	0.332 ±0.061	0.293 ±0.073	0.0004
CpG_8	94,820,244	-311	0.092 ±0.034	0.079 ±0.037	0.0171
CpG_9	94,820,257	-298	0.233 ±0.052	0.189 ±0.054	<0.0001
CpG_10	94,820,259	-296	0.157 ±0.045	0.128 ±0.062	0.0004
CpG_11	94,820,268	-287	0.318 ±0.059	0.283 ±0.063	0.0011
CpG_12	94,820,283	-272	0.305 ±0.058	0.266 ±0.071	0.0002
CpG_13	94,820,289	-266	0.324 ±0.051	0.283 ±0.066	<0.0001
CpG_14	94,820,296	-259	0.343 ±0.059	0.306 ±0.066	0.0001
CpG_15	94,820,310	-245	0.155 ±0.045	0.133 ±0.059	0.0091
CpG_16	94,820,312	-243	0.171 ±0.047	0.151 ±0.062	0.0196
CpG_17	94,820,318	-237	0.160 ±0.044	0.131 ±0.049	0.0003
CpG_18	94,820,327	-228	0.287 ±0.057	0.256 ±0.071	0.0135
CpG_19	94,820,331	-224	0.093 ±0.037	0.071 ±0.038	0.0004
CpG_20	94,820,337	-218	0.183 ±0.056	0.143 ±0.063	0.0001
CpG_21	94,820,341	-214	0.131 ±0.042	0.099 ±0.052	0.0001
CpG_22	94,820,343	-212	0.113 ±0.043	0.098 ±0.041	0.0985
CpG_23	94,820,348	-207	0.233 ±0.057	0.196 ±0.074	0.0003
CpG_24	94,820,366	-189	0.119 ±0.039	0.090 ±0.049	0.0001
CpG_25	94,820,371	-184	0.232 ±0.052	0.200 ±0.058	0.0004
CpG_26	94,820,376	-179	0.159 ±0.049	0.128 ±0.052	0.0001

CpG_27	94,820,379	-176	0.246 ±0.051	0.219 ±0.064	0.0039
CpG_28	94,820,408	-147	0.128 ±0.044	0.105 ±0.054	0.0030
CpG_29	94,820,414	-141	0.135 ±0.040	0.123 ±0.061	0.1480
CpG_30	94,820,416	-139	0.152 ±0.048	0.144 ±0.060	0.3393
CpG_31	94,820,419	-136	0.197 ±0.052	0.171 ±0.063	0.0038
CpG_32	94,820,429	-126	0.277 ±0.062	0.235 ±0.066	<0.0001
CpG_33	94,820,449	-106	0.208 ±0.049	0.167 ±0.061	<0.0001
CpG_34	94,820,456	-99	0.264 ±0.061	0.233 ±0.071	0.0021
CpG_35	94,820,477	-78	0.349 ±0.062	0.311 ±0.069	0.0011
CpG_36	94,820,482	-73	0.142 ±0.044	0.112 ±0.049	0.0006
CpG_37	94,820,494	-71	0.098 ±0.038	0.079 ±0.042	0.0050
CpG_38	94,820,500	-55	0.191 ±0.052	0.155 ±0.054	0.0001
CpG_39	94,820,505	-50	0.161 ±0.045	0.128 ±0.052	0.0002
CpG_40	94,820,534	-21	0.119 ±0.044	0.099 ±0.060	0.0280
CpG_41	94,820,543	-12	0.218 ±0.054	0.197 ±0.063	0.0425
CpG_42	94,820,564	+10	0.093 ±0.036	0.075 ±0.040	0.0011
CpG_43	94,820,588	+34	0.326 ±0.058	0.281 ±0.067	0.0001
Mean			0.204 ±0.039	0.175 ±0.046	<0.0001

[a]Indicates the mean ± standard deviation. [b]The *p*-value was calculated by general linear model adjusted as sex, age, smoking, drinking, BMI, and WHR.

Table S2. Correlation between methylation level of each CpG in the CYP26C1 promoter region and blood parameters.

ID	Loci	Transcriptional start site	Normal								SVO							
			WBC		Hct		LDH		Na		WBC		Hct		LDH		Na	
			r	*p*	*r*	*p*	*r*	*p*	*r*	*p*	*r*	*p*	*r*	*p*	*r*	*p*	*r*	*p*
CpG_2	94,820,183	-372	-0.198	0.034	-0.162	0.083	-0.145	0.126	0.232	0.013	-0.231	0.086	-0.114	0.403	-0.268	0.104	0.324	0.017
CpG_3	94,820,189	-366	-0.197	0.035	-0.243	0.009	-0.248	0.008	0.233	0.012	-0.229	0.090	-0.261	0.052	-0.174	0.297	0.110	0.429
CpG_4	94,820,205	-350	-0.156	0.095	-0.102	0.280	-0.087	0.361	0.075	0.429	-0.192	0.157	-0.094	0.492	-0.173	0.299	0.320	0.018
CpG_5	94,820,209	-346	-0.138	0.141	-0.118	0.210	-0.188	0.046	0.093	0.323	-0.305	0.022	0.006	0.964	-0.203	0.222	0.211	0.125
CpG_6	94,820,231	-324	-0.085	0.365	-0.083	0.377	-0.223	0.018	0.168	0.072	-0.333	0.012	-0.245	0.068	-0.418	0.009	0.155	0.263
CpG_7	94,820,236	-319	-0.157	0.095	-0.141	0.132	-0.273	0.003	0.137	0.144	-0.402	0.002	-0.304	0.023	-0.324	0.047	0.244	0.075
CpG_8	94,820,244	-311	-0.061	0.515	0.047	0.616	-0.221	0.019	0.136	0.149	-0.183	0.177	-0.360	0.006	-0.094	0.575	0.083	0.550
CpG_9	94,820,257	-298	-0.249	0.007	-0.032	0.738	-0.118	0.213	0.150	0.110	-0.319	0.017	-0.122	0.369	-0.144	0.387	0.141	0.311
CpG_10	94,820,259	-296	-0.337	0.000	-0.111	0.238	-0.208	0.027	0.094	0.315	-0.274	0.041	-0.168	0.215	-0.362	0.025	0.245	0.074
CpG_11	94,820,268	-287	-0.251	0.007	-0.178	0.058	-0.075	0.430	0.048	0.612	-0.372	0.005	-0.097	0.478	-0.399	0.013	0.175	0.205
CpG_12	94,820,283	-272	-0.274	0.003	-0.242	0.009	-0.189	0.045	0.206	0.028	-0.368	0.005	-0.021	0.878	-0.203	0.221	0.255	0.063
CpG_13	94,820,289	-266	-0.221	0.018	-0.054	0.570	-0.214	0.023	0.103	0.273	-0.393	0.003	-0.037	0.787	-0.258	0.118	0.300	0.027

CpG_14	94,820,296	-259	-0.174	0.063	-0.105	0.263	-0.274	0.003	0.165	0.079	-0.328	0.014	-0.144	0.290	-0.266	0.106	0.209	0.130
CpG_15	94,820,310	-245	-0.245	0.008	-0.151	0.108	-0.279	0.003	0.225	0.016	-0.361	0.006	-0.265	0.049	-0.173	0.300	0.358	0.008
CpG_16	94,820,312	-243	-0.173	0.064	-0.188	0.045	-0.369	0.000	0.316	0.001	-0.309	0.021	-0.094	0.490	-0.213	0.199	0.202	0.143
CpG_17	94,820,318	-237	-0.225	0.016	-0.197	0.034	-0.176	0.062	0.111	0.239	-0.404	0.002	-0.215	0.112	-0.335	0.040	0.191	0.166
CpG_18	94,820,327	-228	-0.268	0.004	-0.256	0.006	-0.116	0.221	0.084	0.374	-0.326	0.014	-0.207	0.126	-0.357	0.028	0.366	0.006
CpG_19	94,820,331	-224	-0.320	0.000	-0.182	0.052	-0.246	0.009	0.084	0.374	-0.238	0.078	-0.012	0.932	-0.208	0.211	0.131	0.347
CpG_20	94,820,337	-218	-0.260	0.005	-0.215	0.021	-0.139	0.141	0.130	0.165	-0.291	0.029	-0.228	0.091	-0.284	0.084	0.129	0.354
CpG_21	94,820,341	-214	-0.224	0.016	-0.130	0.167	-0.113	0.232	0.110	0.244	-0.200	0.139	-0.227	0.093	-0.151	0.365	0.167	0.228
CpG_22	94,820,343	-212	-0.127	0.175	-0.156	0.095	-0.211	0.025	0.192	0.040	-0.172	0.204	-0.212	0.116	-0.393	0.015	0.225	0.103
CpG_23	94,820,348	-207	-0.190	0.042	-0.191	0.041	-0.143	0.132	0.208	0.026	-0.229	0.090	-0.168	0.215	-0.356	0.028	0.179	0.196
CpG_24	94,820,366	-189	-0.198	0.034	-0.071	0.450	-0.222	0.018	0.192	0.040	-0.297	0.026	-0.307	0.021	-0.310	0.058	0.163	0.238
CpG_25	94,820,371	-184	-0.181	0.053	-0.179	0.056	-0.206	0.028	0.145	0.121	-0.341	0.010	-0.122	0.369	-0.320	0.050	0.298	0.029
CpG_26	94,820,376	-179	-0.209	0.025	-0.075	0.425	-0.100	0.293	0.154	0.100	-0.291	0.029	-0.210	0.119	-0.286	0.082	0.074	0.593
CpG_27	94,820,379	-176	-0.174	0.062	-0.110	0.240	-0.111	0.241	0.130	0.167	-0.244	0.070	-0.144	0.288	-0.326	0.045	0.226	0.101
CpG_28	94,820,408	-147	-0.256	0.006	-0.189	0.043	-0.138	0.146	0.117	0.211	-0.188	0.164	-0.163	0.229	-0.126	0.451	0.139	0.315
CpG_29	94,820,414	-141	-0.254	0.006	-0.161	0.086	-0.136	0.152	0.133	0.157	-0.333	0.012	-0.243	0.071	-0.316	0.054	0.079	0.569
CpG_30	94,820,416	-139	-0.182	0.051	-0.168	0.072	-0.113	0.235	0.152	0.106	-0.327	0.014	-0.279	0.037	-0.236	0.154	0.105	0.449

CpG_31	94,820,419	-136	-0.165	0.077	-0.151	0.108	-0.237	0.011	0.211	0.024	-0.388	0.003	-0.280	0.037	-0.257	0.119	0.128	0.355
CpG_32	94,820,429	-126	-0.272	0.003	-0.152	0.106	-0.157	0.097	0.108	0.251	-0.317	0.017	-0.205	0.129	-0.247	0.134	0.110	0.429
CpG_33	94,820,449	-106	-0.189	0.043	-0.064	0.495	-0.249	0.008	0.150	0.110	-0.265	0.049	-0.147	0.280	-0.157	0.346	0.038	0.783
CpG_34	94,820,456	-99	-0.169	0.071	-0.133	0.155	-0.162	0.086	0.069	0.466	-0.276	0.039	-0.169	0.213	-0.393	0.015	0.170	0.220
CpG_35	94,820,477	-78	-0.219	0.019	-0.195	0.037	-0.043	0.652	0.076	0.421	-0.360	0.006	-0.347	0.009	-0.307	0.061	0.118	0.394
CpG_36	94,820,482	-73	-0.291	0.002	-0.226	0.015	-0.124	0.191	0.196	0.036	-0.381	0.004	-0.183	0.176	-0.306	0.062	0.135	0.332
CpG_37	94,820,494	-71	-0.254	0.006	-0.087	0.358	-0.151	0.110	0.184	0.049	-0.302	0.023	-0.080	0.558	-0.084	0.615	0.042	0.763
CpG_38	94,820,500	-55	-0.234	0.012	-0.157	0.093	-0.186	0.048	0.107	0.253	-0.303	0.023	-0.308	0.021	-0.312	0.057	0.253	0.065
CpG_39	94,820,505	-50	-0.252	0.007	-0.293	0.002	-0.172	0.068	0.190	0.042	-0.324	0.015	-0.335	0.012	-0.229	0.166	0.111	0.425
CpG_40	94,820,534	-21	-0.185	0.048	-0.099	0.293	-0.146	0.122	0.250	0.007	-0.246	0.068	-0.276	0.039	-0.305	0.062	0.171	0.216
CpG_41	94,820,543	-12	-0.117	0.213	-0.171	0.068	-0.013	0.889	0.063	0.503	-0.329	0.013	-0.446	0.001	-0.298	0.069	0.215	0.118
CpG_42	94,820,564	+10	-0.243	0.009	-0.005	0.954	-0.247	0.008	0.115	0.221	-0.286	0.032	-0.301	0.024	-0.236	0.154	-0.031	0.823
CpG_43	94,820,588	+34	-0.218	0.019	-0.241	0.009	-0.146	0.123	0.197	0.035	-0.145	0.288	-0.138	0.310	-0.303	0.064	0.042	0.765
Average			-0.270	0.004	-0.193	0.039	-0.221	0.019	0.191	0.041	-0.377	0.004	-0.247	0.066	-0.334	0.040	0.228	0.098

The *p*-value was calculated by binary person's correlation.

Table S3. Predictive binding sites of transcription factors in the CYP26C1 promoter region.

Transcription Factor	position from transcription start site		String	Dissimilarity (%)
	Start position	End position		
GR-alpha [T00337]	-396	-392	AAAGG	0.21
AP-2alphaA [T00035]	-396	-391	AAAGGC	5.10
GR-alpha [T00337]	-389	-385	CCTAG	8.07
GR-alpha [T00337]	-379	-375	CGAGG	8.28
Sp1 [T00759]	-377	-368	AGGGCGGGGA	1.39
Pax-5 [T00070]	-376	-370	GGGCGGG	1.54
p53 [T00671]	-376	-370	GGGCGGG	3.38
E2F-1 [T01542]	-374	-367	GCGGGGAC	9.03
TFII-I [T00824]	-370	-365	GGACGG	9.51
GR-alpha [T00337]	-363	-359	AGAGG	0.21
RXR-alpha [T01345]	-344	-338	GGGTGGG	5.27
Pax-5 [T00070]	-340	-334	GGGCCAG	1.54
p53 [T00671]	-340	-334	GGGCCAG	4.34
GR-alpha [T00337]	-337	-333	CCAGG	8.07
Pax-5 [T00070]	-323	-317	GGGCGCA	9.55
p53 [T00671]	-323	-317	GGGCGCA	6.40
C/EBPbeta [T00581]	-319	-316	GCAA	0.00
Pax-5 [T00070]	-318	-312	CAAGCCC	0.00
p53 [T00671]	-318	-312	CAAGCCC	3.75
c-Jun [T00133]	-312	-306	CGGGTCA	7.18

RXR-alpha [T01345]	-311	-305	GGGTCAG	1.70
ER-alpha [T00261]	-310	-306	GGTCA	0.00
c-Myb [T00137]	-309	-302	GTCAGTTC	4.75
GCF [T00320]	-305	-297	GTTCTGCGC	7.19
FOXP3 [T04280]	-305	-300	GTTCTG	9.51
FOXP3 [T04280]	-296	-291	GTTGGG	9.51
C/EBPbeta [T00581]	-295	-292	TTGG	1.64
RAR-beta [T00721]	-294	-285	TGGGTTCGCC	2.14
RXR-alpha [T01345]	-293	-287	GGGTTCG	0.85
p53 [T00671]	-290	-284	TTCGCCC	5.35
Egr-3 [T00243]	-290	-278	TTCGCCCACTTT C	6.82
TFIID [T00820]	-281	-275	TTTCTGA	8.01
AP-2alphaA [T00035]	-272	-267	GCCTGC	0.00
GR-alpha [T00337]	-271	-267	CCTGC	8.07
E2F-1 [T01542]	-261	-254	GCGGGGAA	5.48
RelA [T00594]	-260	-250	CGGGGAAGCTC	4.91
NF-kappaB [T00590]	-259	-248	GGGGAAGCTCTG	9.71
NF-kappaB1 [T00593]	-259	-249	GGGGAAGCTCT	2.90
c-Ets-1 [T00112]	-259	-253	GGGGAAG	5.56
STAT4 [T01577]	-257	-252	GGAAGC	4.41
E2F [T00221]	-251	-242	TCTGGCGCGA	9.24
ENKTF-1 [T00255]	-249	-242	TGGCGCGA	6.94
RAR-beta [T00721]	-246	-237	CGCGAACCCG	5.39

RXR-alpha [T01345]	-244	-238	CGAACCC	0.85
GR-alpha [T00337]	-237	-233	GCAGG	8.07
AP-2alphaA [T00035]	-237	-232	GCAGGC	0.00
Pax-5 [T00070]	-236	-230	CAGGCCC	0.00
p53 [T00671]	-236	-230	CAGGCCC	4.13
Sp1 [T00759]	-233	-224	GCCCCGCCCG	0.95
Pax-5 [T00070]	-231	-225	CCCGCCC	1.54
p53 [T00671]	-231	-225	CCCGCCC	3.38
GR-alpha [T00337]	-225	-221	CGAGG	8.28
ETF [T00270]	-218	-208	GCTCGCGGGGC	7.87
Pax-5 [T00070]	-211	-205	GGGCGAG	3.08
p53 [T00671]	-211	-205	GGGCGAG	3.59
Pax-5 [T00070]	-208	-202	CGAGCCC	0.00
p53 [T00671]	-208	-202	CGAGCCC	5.02
ETF [T00270]	-205	-195	GCCCCCAGCCC	5.25
Pax-5 [T00070]	-201	-195	CCAGCCC	0.00
p53 [T00671]	-201	-195	CCAGCCC	3.75
Sp1 [T00759]	-189	-180	GAGCCGCCCC	3.38
Pax-5 [T00070]	-187	-181	GCCGCCC	9.55
p53 [T00671]	-187	-181	GCCGCCC	6.19
GCF [T00320]	-183	-175	CCCCGGCGC	1.07
AP-2alphaA [T00035]	-176	-171	GCCTGC	0.00
GR-alpha [T00337]	-175	-171	CCTGC	8.07
TFII-I [T00824]	-170	-165	CACTCC	6.58

GR-alpha [T00337]	-165	-161	CCTAC	8.07
Pax-5 [T00070]	-158	-152	AGAGCCC	4.01
p53 [T00671]	-158	-152	AGAGCCC	8.54
GR-alpha [T00337]	-152	-148	CCTCC	8.28
GCF [T00320]	-141	-133	GCGCCGCTG	6.99
Pax-5 [T00070]	-136	-130	GCTGCCC	8.01
p53 [T00671]	-136	-130	GCTGCCC	2.81
AP-2alphaA [T00035]	-126	-121	GCCTCC	1.87
GR-alpha [T00337]	-125	-121	CCTCC	8.28
NF-Y [T00150]	-125	-118	CCTCCAAT	5.09
C/EBPalpha [T00105]	-123	-117	TCCAATC	5.57
C/EBPbeta [T00581]	-122	-119	CCAA	1.64
NFI/CTF [T00094]	-122	-115	CCAATCAC	5.56
GR-beta [T01920]	-120	-116	AATCA	4.20
T3R-beta1 [T00851]	-118	-110	TCACCACTC	3.37
MEF-2A [T01005]	-100	-90	CGCTTTAAATA	9.22
HNF-3alpha [T02512]	-97	-90	TTTAAATA	4.84
GR-beta [T01920]	-93	-89	AATAT	3.36
LEF-1 [T02905]	-90	-83	ATGCAAAG	8.76
TCF-4E [T02878]	-89	-83	TGCAAAG	3.15
C/EBPbeta [T00581]	-88	-85	GCAA	0.00
GR [T05076]	-87	-81	CAAAGAC	5.21
CREB [T00163]	-83	-75	GACACGTCA	3.81
ATF-2 [T00167]	-83	-74	GACACGTCAC	2.80

c-Jun [T00133]	-81	-75	CACGTCA	9.01
FOXP3 [T04280]	-73	-68	GTTGTG	0.00
C/EBPbeta [T00581]	-72	-69	TTGT	0.00
VDR [T00885]	-71	-63	TGTGTGAAC	4.62
PXR-1:RXR-alpha [T05671]	-67	-60	TGAACCGG	4.21
TFII-I [T00824]	-60	-55	GGATCG	9.51
TFII-I [T00824]	-56	-51	CGGTCC	9.51
GR-alpha [T00337]	-50	-46	GTAGG	8.07
NF-1 [T00539]	-42	-35	GGAGCCAA	6.95
CTF [T00174]	-41	-30	GAGCCAATATCT	7.45
NF-Y [T00150]	-41	-34	GAGCCAAT	2.13
C/EBPalpha [T00105]	-39	-33	GCCAATA	5.78
C/EBPbeta [T00581]	-38	-35	CCAA	1.64
GATA-2 [T00308]	-38	-30	CCAATATCT	7.78
GR-beta [T01920]	-36	-32	AATAT	3.36
GATA-1 [T00306]	-34	-29	TATCTA	0.28
TBP [T00794]	-32	-23	TCTATATAAA	1.87
FOXP3 [T04280]	-27	-22	ATAAAC	9.51
AR [T00040]	-24	-16	AACGTGTCC	8.33
TFII-I [T00824]	-21	-16	GTGTCC	9.51
NF-1 [T00539]	-11	-4	TGGGCCAA	0.00
Pax-5 [T00070]	-10	-4	GGGCCAA	9.55
p53 [T00671]	-10	-4	GGGCCAA	6.10

C/EBPbeta [T00581]	-7	-4	CCAA	1.64
